# The rubber hand illusion induced by visual-thermal stimulation

**DOI:** 10.1038/s41598-018-29860-2

**Published:** 2018-08-20

**Authors:** Jörg Trojan, Xaver Fuchs, Sophie-Louise Speth, Martin Diers

**Affiliations:** 1 University of Koblenz–Landau, Campus Landau, Department of Psychology, Fortstraße 7, 76829 Landau, Germany; 2 Bielefeld University, Faculty of Psychology and Sports Science, Biopsychology & Cognitive Neuroscience, Universitätsstraße 25, 33615 Bielefeld, Germany; 3 Ruhr-University Bochum, LWL University Hospital, Department of Psychosomatic Medicine and Psychotherapy, Alexandrinenstraße 1–3, 44791 Bochum, Germany; 4 Department of Cognitive and Clinical Neuroscience, Central Institute of Mental Health/Medical Faculty Mannheim, Heidelberg University, Mannheim, Germany

## Abstract

In the rubber hand illusion (RHI), synchronous touch of a real hand and an artificial hand leads to the feeling of the artificial hand belonging to one’s own body. This study examined whether the RHI can be induced using visual–thermal instead of visual–tactile stimulus patterns and to which extent the congruency between temperature and colour of the visual stimulus influences the RHI. In a within-subject design, we presented cold vs. warm thermal stimuli to the participants’ hidden hand combined with red vs. blue visual stimuli presented synchronously vs. asynchronously at a fake hand. The RHI could be induced using visual–thermal stimuli, yielding RHI vividness ratings comparable to the visual-tactile variant. Congruent (warm–red, cold–blue) synchronous stimulus patterns led to higher RHI vividness than incongruent (warm–blue, cold–red) synchronous combinations; in the asynchronous conditions, an inverse effect was present. Temperature ratings mainly depended on the actual stimulus temperature and were higher with synchronous vs. asynchronous patterns; they were also slightly higher with red vs. blue light, but there were no interactions with temperature or synchrony. In conclusion, we demonstrated that the RHI can be induced via visual-thermal stimuli, opening new perspectives in research on multi-sensory integration and body representations.

## Introduction

The so-called rubber hand illusion (RHI) has become a standard paradigm for studying the plasticity of body representations. The RHI was first demonstrated by Botvinick & Cohen^1^, who observed that repetitive synchronous touch of a real hand and an artificial hand led their participants to mislocalise the touch to the artificial hand and even induced feelings of the artificial hand belonging to their body. Since this original report, the paradigm has been used in many contexts and variants^2^, including the induction of out-of-body illusions^3,4^ and applications in animal experiments^5,6^.

Usually, the RHI is induced with soft brushes, i.e. via visual-tactile integration. However, the RHI does not depend on any specific sensory modality or mode of stimulation. Some studies have used ‘tap’-like tactile stimuli^7^ or even sharp needles^8^ instead of brushstrokes. Furthermore, the RHI can also be induced via synchronous movements of the participant’s own and the artificial hand^9–11^ as well as—without any visual feedback—by synchronising the participant’s touch movement to an artificial hand with touch stimuli delivered to his or her own hand^12^. The RHI can also be induced by having participants just watch a visual stimulus move over an artificial hand without delivering any stimulation to their real hand^13^. In some participants, the RHI even occurs spontaneously by watching an artificial hand, without any stimulation^14^.

To date, only few studies have addressed the potential role of interoception in the context of the RHI. Interoception can be conceptualised as “the sense of the physiological condition of the body”^15,16^, tightly coupled with homeostatic functions and encompassing not only visceroception but also our senses of temperature, pain, and even “affective” touch. A recent study introduced a “cardiac” RHI, in which visual stimuli at a virtual hand were synchronised with the participants’ heartbeat^17^. Optimising stimulus characteristics for “affective” touch fibres in the skin has been reported to increase the RHI^18^. Some researchers have also observed reduced skin temperature during the RHI^19^ and, vice versa, cooling of the hand to ease the induction of the RHI^20^. These findings suggested that body representations are possibly coupled to fundamental brain-stem mechanisms such as thermoregulation, but recent studies have cast doubt on their replicability^14,21^.

In the present study, we set out to investigate whether the RHI can be systematically induced via visual-thermal stimulus patterns and whether this effect shows an interaction between the quality of the thermal stimulus—warm vs. cold—and the colour of the visual stimulus—red vs. blue. The latter point was inspired by anecdotal evidence on blue visual stimuli inducing “cooler” sensations than red visual stimuli when presented during the RHI^13^.

The hue–heat hypothesis^22^ posits that we spontaneously associate red with warmth and blue with cold. This has been demonstrated, for instance, with shorter reaction times to congruent (warm–red, cold–blue) compared to incongruent (warm–blue, cold–red) combinations of colours and temperature words in an implicit association test, as well as in discrimination tasks using colours with temperature words and actual thermal stimuli^23^. Red light also leads to thermal stimuli being perceived as warmer and more painful compared to blue light^24,25^.

**Figure 1:**
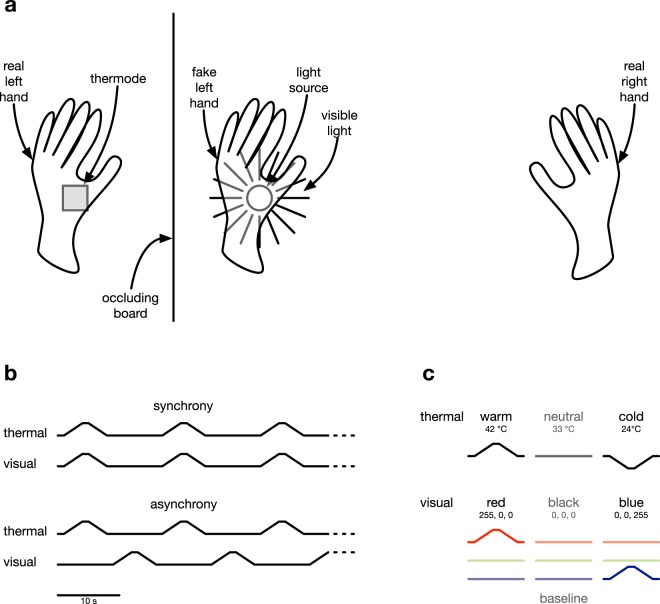
(**a**) Experimental setup. Participants sat in front of a table and placed their left hand on a thermode, set into the table surface. The left hand was occluded from sight by a vertical board. The fake hand was placed at a distance of 15 cm to the right of the left hand. Through a hole in the table, light could be projected to the underside of the fake hand. (**b**) Synchronous and asynchronous stimulation. All stimuli had a duration of 7 s, consisting of ramps increasing/decreasing (3 s) towards the target temperature (1 s) and returning to baseline (3 s). In the synchrony condition, a total of 16 synchronous bimodal visual–thermal stimuli were presented with inter-stimulus intervals of 9 s. In the asynchrony condition, unimodal visual and thermal stimuli were presented alternately with inter-stimulus intervals of 1 s. (**c**) Stimulus parameters. The baseline temperature was set to 33 °C; warm stimuli had a peak temperature of 42 °C; cold stimuli had a minimum of 24 °C. The baseline colour was black (RGB: 0; 0; 0) and the two target colours were red (RGB: 255; 0; 0) and blue (RGB: 0; 0; 255).

Based on these findings, we hypothesised that congruent visual-thermal stimulus pairs (warm–red, cold–blue) should lead to a stronger RHI than incongruent pairs (warm–blue, cold–red). We tested this using a within-subject design with the factors synchrony (synchrony vs. asynchrony of thermal stimuli to the left hand and light stimuli to the rubber hand), temperature (warming vs. cooling of the thermal stimulus presented to the hand), and colour (red vs. blue light). See Fig. 1 for an overview of the experimental setup.

## Results

### Temperature ratings

**Figure 2:**
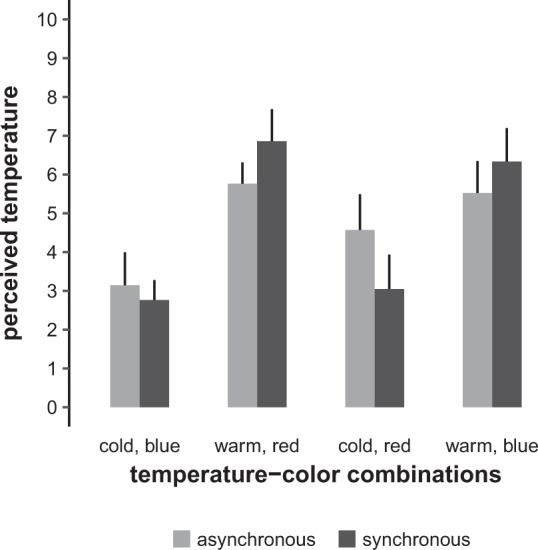
Perceived temperature of the thermal stimulus (numerical rating scale, 0–10) in the eight different combinations of temperature, colour, and synchrony. The graph shows the mean ratings of all participants with 95% confidence intervals.

We first checked the validity of our experimental manipulations by examining their effects on the perceived temperature of the stimuli using a linear mixed model analysis with the factors synchrony, temperature, and colour (model type A, see Methods for details). See Fig. 2 for a graphical representation of the results. As expected, the perceived temperature depended strongly on the actual temperature (F_1,140_ = 95.2, p < 0.0001), but not on synchrony (F_1,140_ = 0.0, p = 1.0000). Furthermore, a significant main effect of colour (F_1,140_ = 4.9, p = 0.0290) was present, with red stimuli being rated slightly warmer than blue stimuli. These results show that warm and cold stimuli were suitable for inducing the intended subjective percepts and that, in line with the hue–heat hypothesis, a small but detectable influence of colour was present. Following up on the significant synchrony × temperature interaction (F_1,140_ = 11.5, p = 0.0009), we conducted a complete set of six pairwise post-hoc comparisons of the four possible combinations, which all yielded significant results (see Supplementary Information). In particular, the results indicated that warm stimuli were rated warmer and cold stimuli were rated colder when the thermal and visual stimuli were presented synchronously compared to asynchronously. It is noteworthy that we did not find a significant synchrony × temperature × colour interaction which is at odds with the hue–heat hypothesis.

### Illusion vividness ratings

**Figure 3:**
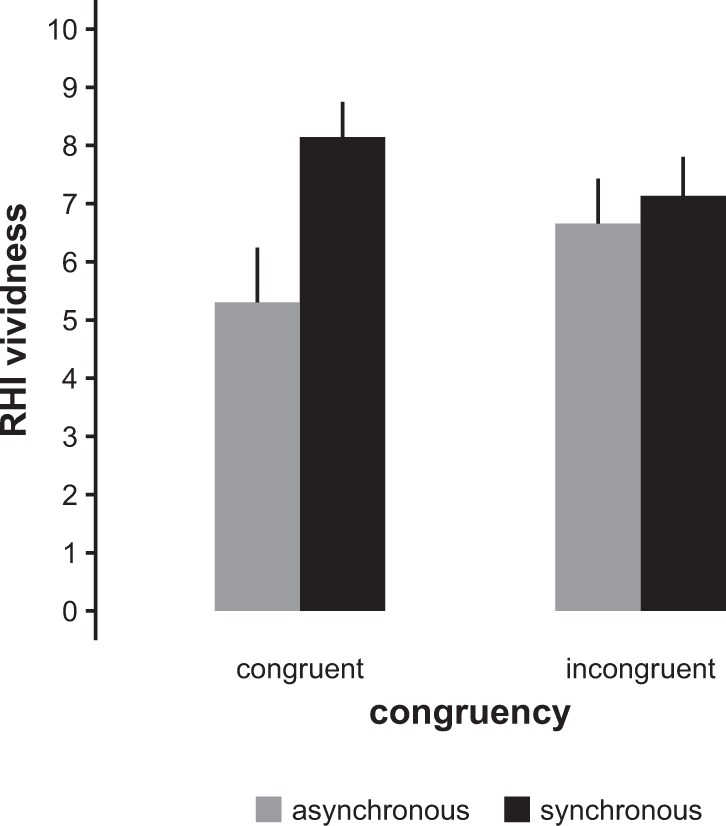
RHI vividness score (numerical rating scale, 0–10) in the four different combinations of synchrony and congruency. The graph shows the mean scores of all participants with 95% confidence intervals.

Our main hypothesis was tested using a linear mixed model estimating the effects of the factors synchrony and congruency on the vividness of the RHI (model type B, see Methods). See Fig. 3 for a graphical representation of the results. We found a significant main effect for synchrony (F_1,145.97_ = 33.4, p < 0.0001) on vividness, with synchronous stimulation leading to higher scores than asynchronous stimulation. As predicted, we also found a significant synchrony × congruency interaction (F_1,145.97_ = 15.6, p = 0.0001). With one exception, all pairwise post-hoc comparisons of this interaction yielded significant results (see Supplementary Information): In incongruent (warm–blue, cold–red) patterns, no significant difference between asynchronous and synchronous stimulation was present, while this was clearly the case in congruent (warm–red, cold–blue) patterns. Interestingly, RHI vividness was not only lower with incongruent compared to congruent synchronous stimulation; it was also higher with incongruent compared to congruent asynchronous stimulation (see Fig. 3). While we had not predicted such a pattern, it is plausible that congruent asynchronous stimulation should lead to larger interference with the illusion than incongruent asynchronous stimuli.

**Figure 4:**
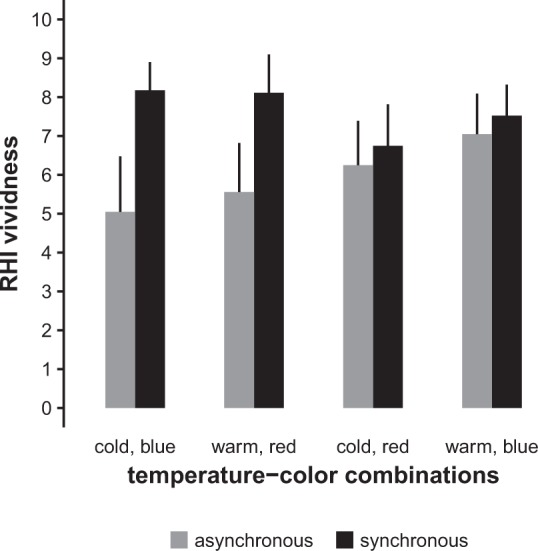
RHI vividness score (numerical rating scale, 0–10) in the eight different combinations of synchrony, temperature, and colour. The graph shows the mean scores of all participants with 95% confidence intervals.

In order to get a better picture of the underlying processes, we determined the distinct contributions of temperature and colour using the 2 × 2 × 2 type A model (see Methods). See Fig. 4 for a graphical representation of the results. This analysis reproduced the highly significant effect of synchrony (F_1,145.96_ = 34.9, p < 0.0001) already found in the 2 × 2 type B model. The significant synchrony × temperature × colour interaction (F_1,145.96_ = 16.0, p < 0.0001) mainly encompasses the synchrony × congruency interaction from the 2 × 2 type B model: With synchronous stimulation, congruent combinations yielded higher RHI vividness ratings than incongruent combinations; vice versa, with asynchronous stimulation, incongruent combinations yielded higher RHI vividness ratings than congruent combinations. This model also revealed a trend for a main effect of temperature (*F*_1,145.96_ = 3.5, p = 0.0644), with warmer temperatures leading to higher RHI vividness. However, separate follow-up analyses for the synchronous and asynchronous conditions showed that this observation should not be overinterpreted: The effect was even less prominent in the asynchronous condition alone (*F*_1,62.04_ = 3.1, p = 0.0842) and not significant at all in the synchronous condition (*F*_1,63.00_ = 1.3, p = 0.2594).

**Figure 5:**
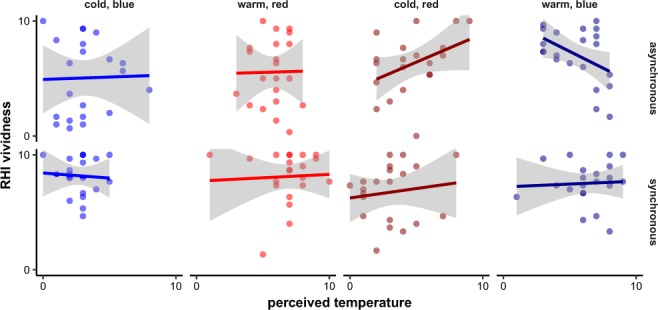
The dependence of RHI vividness scores (numerical rating scale, 0–10) on perceived temperature (numerical rating scale, 0–10) in the eight different combinations of temperature, colour, and synchrony.

Despite identical physical temperature the subjective rating of the stimuli varied largely (see above), so we wondered whether these differences might have influenced the vividness of the illusion. Figure 5 shows the relationship between perceived temperature and RHI vividness, separately for each design cell of the experiment. If at all, only weak relationships between perceived temperature and RHI vividness were present in the congruent (cold–blue, warm–red) synchronous and asynchronous conditions as well as in the incongruent (cold–red, warm–blue) synchronous conditions. In the incongruent asynchronous conditions, however, perceived temperature ratings partly predicted the RHI vividness (linear regressions; cold–red: F_1,18_ = 3.5, p = 0.0775; warm–blue: F_1,19_ = 5.0, p = 0.0371) with a peculiar pattern: Warm stimuli which were rated as rather cold and cold stimuli which were rated as rather warm were associated with high vividness ratings, partly even superseding those reached in the synchronous conditions. This observation highlights yet another facet of the finding that congruent asynchronous stimulation leads to larger interference with the illusion than incongruent asynchronous stimuli (see Fig. 3): This effect is not driven by the absolute temperature, but rather by the subjectively perceived temperature of the stimulus.

## Discussion

This study showed that the RHI can be induced using visual–thermal stimulus patterns. Furthermore, we demonstrated the important role of temperature–colour congruency: As expected, synchronous congruent stimulus patterns led to stronger illusions than synchronous incongruent patterns. In addition, an inverse effect was present for asynchronous stimulation, where congruent patterns led to weaker illusions than incongruent patterns.

The main purpose of this study was to demonstrate the first systematic induction of a visual–thermal RHI. RHI vividness was higher for synchronous compared to asynchronous stimulation, providing the usual indicator for the presence of the illusion. It is noteworthy that with this novel induction method we observed relatively high RHI vividness scores (Figs 3 and 4). Participants had been preselected based on their responses to the ordinary brushstroke method, but above-average susceptibility of our sample to the classical RHI may not have been the only reason for the success of our unusual approach. After all, participants only received a total of 16 stimulus pairs, spread over a duration of roughly four minutes. This is a relatively sparse stimulation pattern compared to previous RHI studies, which typically deliver stimuli at a frequency of at least 0.5 Hz with trial durations of 30 s up to several minutes. The effortless induction of the RHI with visual–thermal stimulation patterns may be partly due to thermal information being relayed via small-diameter Aδ- and C-fibres, which have much lower conduction velocities than tactile Aβ-fibres. As a consequence, thermal stimuli are processed with a considerably lower temporal precision, potentially facilitating integration with other sensory information^26^. In line with these considerations, slow and smooth tactile stimulation aimed at “affective” touch C-fibres also increases the RHI^18^.

Furthermore, we were interested in whether RHI vividness also depended on the congruency between the quality of the thermal stimulus—cold vs. warm—and the colours blue vs. red. Based on previous findings^22–24^, we assumed that red should be associated with higher rather than with lower temperature and, vice versa, that blue should be associated with lower rather than with higher temperature. As a consequence, when presented simultaneously, congruent warm–red and cold–blue stimuli should be more easily linked to each other and thus induce a stronger RHI than incongruent warm–blue and cold–red stimuli. While the expected pattern was present in the synchronous stimulation conditions, we did not find a main effect of congruency, due to the asynchronous conditions showing an effect in the opposite direction. In other words: Congruent stimulation substantially increased RHI vividness in the synchronous conditions but in the asynchronous conditions it was associated with substantially *lower* RHI vividness.

While we had not expected such a strong effect of congruency in the asynchronous condition, it is quite plausible upon closer inspection: In the same manner as the congruent synchronous conditions provide the strongest coupling of the thermal and visual stimuli, the congruent *a*synchronous conditions provide the clearest *de*coupling: Here, the stimuli are presented temporarily separated from each other, counteracting the induction of an RHI. The situation is more ambiguous in the incongruent asynchronous conditions: Due to their incongruency, thermal and visual stimuli may appear as less strongly associated with each other, thereby reducing perceptual conflict and leading to increased RHI vividness in our sample, which consisted of participants who were particularly prone to the illusion. This would be in line with earlier findings showing that the RHI can be induced using visual stimuli alone^13^. In addition, cold–red and warm–blue asynchronous stimuli may have been perceived as more congruous than intended: The neutral baseline temperatures were warmer than the cold stimuli and colder than the warm stimuli. Thus, the incongruent thermal–visual patterns were actually congruent in respect to the relative temperature changes.

As would be expected, the perceived temperature of the stimuli was mainly determined by their physical temperature. We also observed that warm stimuli were rated warmer and cold stimuli were rated colder when the thermal and visual stimuli were presented synchronously rather than asynchronously. This effect is in line with known mechanisms of multisensory integration, namely, that spatially and temporally congruent stimuli “sharpen” the percept and provide a stronger contrast to the baseline^26^. However, we only found limited support for the hue–heat hypothesis^22^: The perceived temperature of red stimuli was rated just slightly higher than that of blue stimuli, on average by 0.6 points of the 0–10 numerical rating scale. More to the point, we would have expected colour to modulate the perception of a given temperature, i.e. a warm–red combination yielding higher perceived temperatures than a warm–blue combination, and a cold–blue combination yielding lower perceived temperatures than a cold–red combination. We found no statistical evidence for such an interaction. As can be seen in Fig. 2, the only obvious irregularity lies in the relatively high perceived temperature in the asynchronous cold–red combination. This finding is puzzling, but if taken for granted, it actually works against the hue–heat hypothesis: It indicates that cold thermal stimuli were perceived as warmer if the visual red stimuli were interspersed with them compared to if they were presented simultaneously, which, again, would be rather in line with an interpretation in terms of multisensory spatiotemporal integration. Another indirect line of reasoning in favour of the hue–heat hypothesis could be based on the lack of difference in the illusion vividness ratings between asynchronous and synchronous stimulation with incongruent (warm–blue, cold–red) temperature–colour combinations. However, this finding may be driven by several other effects as well—e.g. incongruent asynchronous stimuli being perceived as more congruous than intended (see above)—and it remains unclear why a hue–heat effect should be lacking in the explicit temperature rating while being implicitly present in the RHI vividness scores.

The few other studies which have systematically tackled the hue–heat hypothesis to date^22–24^ differ largely in scope and method. We are only aware of one other paper reporting an—at least superficially—similar approach: In that study a −20 °C cold stimulus was presented for 500 ms in combination with blue or red lights and yielded average differences in perceived temperature of roughly 5 on a 0–10 scale similar to ours^24^. At first sight this largely seems to contradict our findings, but it has to be considered that the stimulus used in that study mainly activated nociceptive rather than thermal fibres, predominantly producing hot and cold “burning” sensations, thereby possibly easing the perceptual relabeling of the stimulus based on colour cues, especially at such short presentation times.

As far as we can tell, our study is unique in combining colour and temperature within a single, integrated percept. One could have expected that this should boost a possible effect of colour on temperature perception, especially in the warm–red condition, which appears very similar to known properties of infrared lamps, ceramic stovetops etc. However, we did not find any indication of such an effect; actually, warm–red and warm–blue stimuli were basically rated identically. We conclude that while, according to other studies, colour may influence temperature perception if used as a context, this does not happen if colour is interpreted as an aspect of a thermal stimulus. In other words, in an integrated thermal–visual percept, the thermal aspect “overwrites” visual information.

This is also in line with the results in a paper studying the effect of the classically induced RHI on thermal perception^27^. Participants were asked to rate whether the temperature of a neutral vs. cold thermal stimulus presented to their hand increased or decreased compared to a previously presented neutral vs. cold thermal stimulus while watching neutral vs. cold visual stimuli—a plastic cube vs. an ice cube—synchronously touching the artificial hand. Visual cues only affected perceived temperature change if thermal stimuli were kept constant, but this effect was completely obliterated if thermal stimuli actually changed and thereby provided a more valid source of information for this rating.

Inspired by the analgesic effect of watching one’s own hand^28^, several previous studies examined how the RHI influences thermal pain^29–31^, with mixed findings: Effects on pain ratings could either not be found at all^30^ or were rather small^29^, partly even indicating an increase rather than the expected decrease in pain intensity^29^. Crucially, the analgesic effect during the RHI seems to depend on the spatial congruency between one’s own and the artificial hand^31^. Unfortunately, none of these studies used temperature ratings, but rather assessed a forced-choice judgement of thermal change^27^, pain intensity^29,30^ or pain thresholds^31^, making it hard to draw conclusions on how and to which extent temperature perception *per se* was affected.

As already mentioned above, thermal information is processed differently from touch already in the periphery, and this distinction exists up to the cortical level. Fine touch is processed via Aβ-fibres, spinothalamical projections of the lemniscal system and the thalamus to the primary somatosensory cortex. Small-diameter C- and Aδ-fibres, however, terminate in the superficial layers of the dorsal spinal horn. From there, central projections travel via the anterolateral system, which includes the spinothalamical tract and collaterals to several brainstem and midbrain structures involved in homeostatic control. From the thalamus, projections reach the primary somatosensory cortex, but their main target is the insular cortex. Historically, this area was mainly associated with visceral representations^32^, but recent accounts stress the insula’s central role in a holistic conceptualisation of interoception, underlying body perception, emotion, and, ultimately, the emergence of awareness^15,16,33^.

Only few studies to date have systematically examined the role of interoceptive stimuli in the RHI. Within a standard RHI setup, optimising stimulus characteristics for “affective touch” fibres in the skin—i.e., delivering them slowly with smooth materials—has been reported to increase the illusion^18^. Two very impressive studies show that visual stimuli synchronised with a participant’s heartbeat instead of touch can be used to induce the RHI^17^ and even increase identification with a complete virtual body^34^. There is some evidence that the RHI is accompanied by reduced skin temperature^19^ and, vice versa, cooling of the hand may ease the induction of the RHI^20^, but recent studies have cast doubt on their replicability^14,21^. Finally, lower interoceptive sensitivity measured with a heartbeat detection task has been shown to predict a reduced susceptibility to the RHI^35^. Combined with the evidence from our study, these findings show that interoceptive information is an important constituent of how we perceive our body and should receive more interest in future research.

Previous research has identified several premotor and parietal areas involved in the visual–tactile variant of the RHI^7,12,36^. It is possible, however, that the visual-thermal RHI relies on different or at least additional underlying processes. Thermal information is mainly represented in the insular cortex^37^. On the one hand, it is possible that it is first mapped onto the somatosensory representation in SI and then being integrated using similar processes as in the visual–tactile RHI. On the other hand, it seems equally plausible that visual thermal integration is based directly on insular representations. More to the point, there is evidence that the insula is involved in cross-modal binding, including visual and auditory stimuli^38,39^. To our knowledge, this finding has never been discussed in the context of the RHI, but it opens the possibility of insular involvement in any multi-modal integration process.

In conclusion, our findings show that the RHI can be induced at least equally well using visual-thermal stimulation patterns and that this effect can be boosted by using congruent (warm–red, cold–blue) compared to incongruent (warm–blue, cold–red) colour–temperature combinations. Interestingly, temperature perception was affected by stimulus colour to a minor degree only. This could mean that homeostatically relevant somatosensory information receives stronger weights in the process of multisensory integration than potentially more ambiguous exteroceptive information.

From a methodological perspective, it is also noteworthy that we present the first experimental evidence of a RHI based on non-mechanical somatic stimulation conveyed via small-diameter fibres. This opens new prospects in human and animal research on multi-sensory integration. The possibility to selectively address interoceptive pathways will help shed light on their role in body perception and their potential involvement in neurological and functional disorders.

## Methods

### Ethics

Informed consent was obtained, and the study was approved by the ethics committee of the Medical Faculty Mannheim, Heidelberg University, and adhered to the Declaration of Helsinki. The experiment was performed in accordance with the ethical guidelines of the German Psychological Society (DGPs).

### Participants

Susceptibility to the RHI varies considerably between persons; and in part of the population (approximately 20–30 per cent) it cannot be induced at all. We therefore selected our sample based on a short pretest, administered to more than 50 interested students at the University of Mannheim and at the University of Koblenz–Landau, Campus Landau. Our inclusion criteria consisted of being right-handed according to the Edinburgh Handedness Inventory^40^ and being clearly susceptible to the conventional RHI (see Supplementary Information for details).

Twenty-one participants fulfilled the inclusion criteria. Their mean age was 22.5 years (range 18–39 years), 17 participants were female (81%), all of them were students, 9 studied psychology (43%). The participants either received course credits or a remuneration of 15 €.

### Experimental Design

Our experiment consisted of a 2 × 2 × 2 within-subject design with the factors synchrony (synchrony vs. asynchrony of thermal stimuli to the left hand and light stimuli to the rubber hand), temperature (warming vs. cooling) and colour (red vs. blue light). The resulting 8 conditions were presented in randomised order^41^.

### Apparatus and Protocol

Participants sat in front of a table and placed the thenar of their left hand on a thermode (size 3 cm × 3 cm; Modular Sensory Analyzer, Somedic, Hörby, Sweden), set into the table surface. The left hand was occluded from sight by a vertical board. At a distance of 15 cm to the right of the left hand, a left-hand sex-matched prosthetic glove (Otto Bock, Duderstadt, Germany) was placed. Through a hole in the table, light could be projected to the underside of the rubber hand using a video projector (InFocus IN26+, Model: 260, InFocus, Portland, Oregon, USA). Perceptually, this resulted in light seeping from underneath the rubber hand, focused on its thenar. See Fig. 1a for an overview of the setup.

The baseline temperature of the thermode was set to 33 °C. Warm stimuli had a peak temperature of 42 °C; cold stimuli had a minimum of 24 °C. All thermal stimuli consisted of ramps increasing/decreasing at a rate of 3 °C/s, remaining at the respective target for 1 s, and then returning to the baseline at the same rate, yielding a total stimulus duration of 7 s (Fig. 1c).

The baseline colour was black (RGB: 0; 0; 0) and the two target colours were red (RGB: 255; 0; 0) and blue (RGB: 0; 0; 255). Colour stimuli consisted of increasing the respective RGB colour components at a rate of 85 bits/s, remaining at the target colour for 1 s, and then returning to baseline at the same rate, also yielding a total stimulus duration of 7 s (Fig. 1c).

In the synchrony condition, a total of 16 synchronous bimodal visual–thermal stimuli were presented with inter-stimulus intervals of 9 s. In the asynchrony condition, unimodal visual and thermal stimuli were presented alternately with inter-stimulus intervals of 1 s. In half of the trials, this sequence started with thermal stimuli, in the other half it started with visual stimuli. See Fig. 1b for a graphical depiction of these sequences. The experiment was controlled with a custom-made script using PsychoPy^42^.

### Ratings

After each condition, participants were first asked to rate the perceived temperature of the thermal stimulus on a discrete numerical scale ranging from 0 (“very cold”) to 10 (“very warm”). Then they were given a modified version of the original rubber hand questionnaire^1^ (see Table 1), which had to be rated on a discrete numerical scale ranging from 0 (“not at all”) to 10 (“most intense”).

**Table 1:** RHI experience questionnaire.

During the experiment there were times when	
(1)	It seemed as if I was feeling the cooling/warming of the thermode where I saw the rubber hand lighten up.
(2)	It seemed as though the cooling/warming of the thermode was caused by the lightening of the rubber hand.
(3)	The rubber hand felt as if it was my own hand.
(4)	I felt as if my real hand was drifting to the right (towards the rubber hand).
(5)	It seemed as if I might have more than one right hand or arm.
(6)	It seemed as if the cooling/warming of the thermode came from somewhere between my own hand and the rubber hand.
(7)	It felt as if my real hand was changing into rubber.
(8)	It appeared (visually) as if the rubber hand was drifting towards the left (towards my hand).
(9)	The rubber hand began to look like my own (real) hand in terms of shape, skin colour, freckles or other features.

### Data processing and statistical analyses

In line with usual practice, we averaged the first three items of the rubber hand questionnaire and used this integrated measure as a vividness score of the illusion. See Supplementary Information for details on the individual items.

We analysed our data with two types of statistical models. Type A reproduced the full 2 × 2 × 2 design with the factors synchrony, temperature, and colour. However, main effects of temperature and colour are of minor relevance for our main hypothesis and, more to the point, in this model the effects of interest—the temperature–colour congruency and its interaction with synchrony—are “hidden” in two- and three-way interactions. Therefore, in addition to the full model we used a more appropriate model for testing our main hypothesis. Type B focused on congruent vs. incongruent temperature–colour combinations, i.e. warm–red and cold–blue vs. warm–blue and cold–red, yielding a 2 × 2 design with the factors synchrony and congruency.

Both types of analysis were implemented with linear mixed models. In addition to the 3 resp. 2 fixed within-subject factors, we also included a random factor allowing for inter-individual intercept differences. Details on the model selection procedure, the model specifications, and a comparison of their respective results can be found in the Supplementary Information.

Exploratory analyses of the relationships between variables were conducted with linear regression models.

Graphs show the mean ratings of all participants with error bars indicating the 95% confidence intervals of the standard error of the mean.

All statistical analyses were performed using the R environment for statistical computing and graphics^43^ with the RStudio integrated development environment (RStudio Inc., Boston, MA, USA). Linear mixed models were analysed with lme4 and lmerTest packages^44,45^; post-hoc comparisons were calculated using the emmeans package^46^; graphs were created with the ggplot2 package^47^.

### Data availability

Data and analyses are available at https://osf.io/at5sx.

## Electronic supplementary material

Supplementary Information

Dataset 1
