# Supplementary material for: The rubber hand illusion induced by visual-thermal stimulation
Source: Sci Rep. 2018 Aug 20;8:12417. doi: 10.1038/s41598-018-29860-2 (PMC6102275; doi:10.1038/s41598-018-29860-2)
Supplement: Supplementary file 1 — Supplementary Information [file 41598_2018_29860_MOESM1_ESM.pdf]

The rubber hand illusion induced by visual-thermal stimulation

Supplementary Information

Jörg Trojan, Xaver Fuchs, Sophie-Louise Speth, Martin Diers

Contents

RHI pretest

Detailed RHI questionnaire results

Linear Mixed Model specification

Full data analysis

RHI pretest

The pretest was administered to more than 50 interested students at the University of Mannheim and at the University of Koblenz-Landau, Campus Landau. Using the ordinary brushstroke method, potential participants were stimulated synchronously and asynchronously for 45 s each and rated item 3 from the RHI experience questionnaire, “The rubber hand felt as if it was my own hand”, on a 0–10 numerical scale. Only right-handed participants yielding a value of 4 or more in the synchronous condition and at least two points less in the asynchronous condition were included in the main study.

Detailed RHI questionnaire results

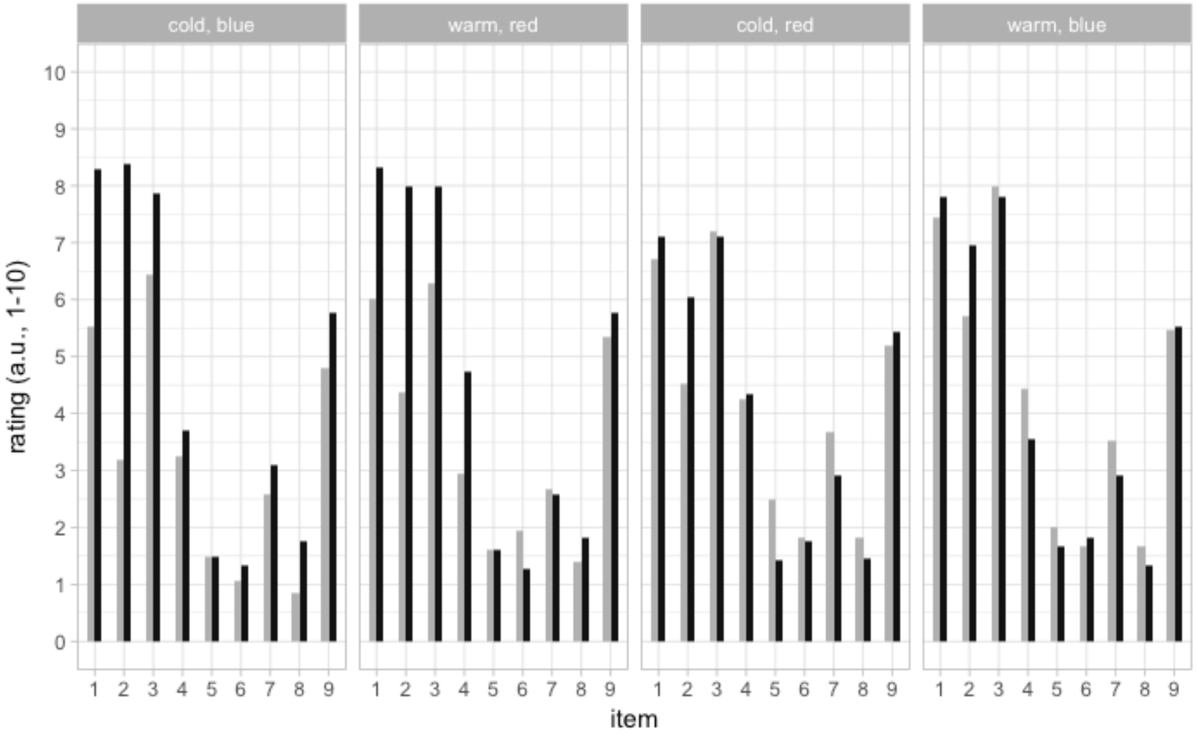

The graph shows mean ratings of all participants by temperature–color combination and synchrony (black – synchrony; grey – asynchrony).

The results show that, as expected, the first three items of the RHI questionnaire were distinguished best between asynchronous and synchronous stimulation. Interestingly, item 2, ("It seemed as though the cooling/warming of the thermode was caused by the lightening of the rubber hand.") provided the best differentiation. In fact, in the two incongruous conditions (cold–red, warm–blue) it was obviously responsible for yielding a synchronous vs. asynchronous difference in the RHI score at all. The "control" items 4–9 yielded no obvious differences, with one exception: Item 4 ("I felt as if my real hand was drifting to the right (towards the rubber hand).") differentiated between synchronous and asynchronous stimulation in the warm–red condition.

Linear Mixed Model specification

Type A reproduced the full 2 × 2 × 2 design with the factors synchrony, temperature, and color. Type B combined congruent vs. incongruent temperature–color combinations, i.e. warm–red and cold–blue vs. warm–blue and cold–red, yielding a 2 × 2 design with the factors synchrony and congruency. In addition to the fixed factors, we included a random factor allowing for inter-individual intercept differences. This was based on our expectation of large inter-individual differences in respect to RHI susceptibility and temperature perception.

The perceived temperature of the stimulus was analyzed using a type A model:

*lmer(perc_temp ~ synchrony * color * temperature + (1 | subj_id), data = data)*

Obviously, we expected a large main effect of temperature, but, according to the hue–heat hypothesis, also a smaller effect of color. A main effect of synchrony was not expected, but we included this factor in order to check for any interactions with color and temperature, which could arise from the differences in the RHI ownership.

Our main hypothesis focused on the effect of visual-thermal congruency as well as its interaction with synchrony on the RHI vividness score. However, in the type A model, congruency cannot be tested as a main effect but only in terms of the two-way color × temperature interaction. Even more complicated, the interaction between congruency and synchrony becomes encapsulated in the three-way synchrony × color × temperature interaction:

*lmer(RHI_vividness ~ synchrony * color * temperature + (1 | subj_id), data = data)*

This means that with the type A model, our effects of interest would have to be implemented as contrasts of two- and three-way interactions, making any tests and

interpretations unnecessarily hard. Therefore, we relabeled all warm–red and cold–blue conditions as "congruent" and all warm–blue and cold–red conditions as "incongruent" and used a type B model for the analysis:

*lmer(RHI_vividness ~ synchrony * congruency + (1 | subj_id), data = data)*

The type A model was fitted anyway in order to check whether the above reasoning was valid and to identify possible unexpected effects. As discussed in the main text, the results from the type A model basically supported the results found with the simplified type B model, but yielded additional limited evidence ($p = 0.07$) for a main effect of temperature on RHI vividness.

The rubber hand illusion induced by visual-thermal stimulation

Statistical analysis and graphs

Jörg Trojan, Xaver Fuchs, Sophie-Louise Speth, Martin Diers

10 May 2018

Temperature by by synchrony, color and temperature

Boxplot

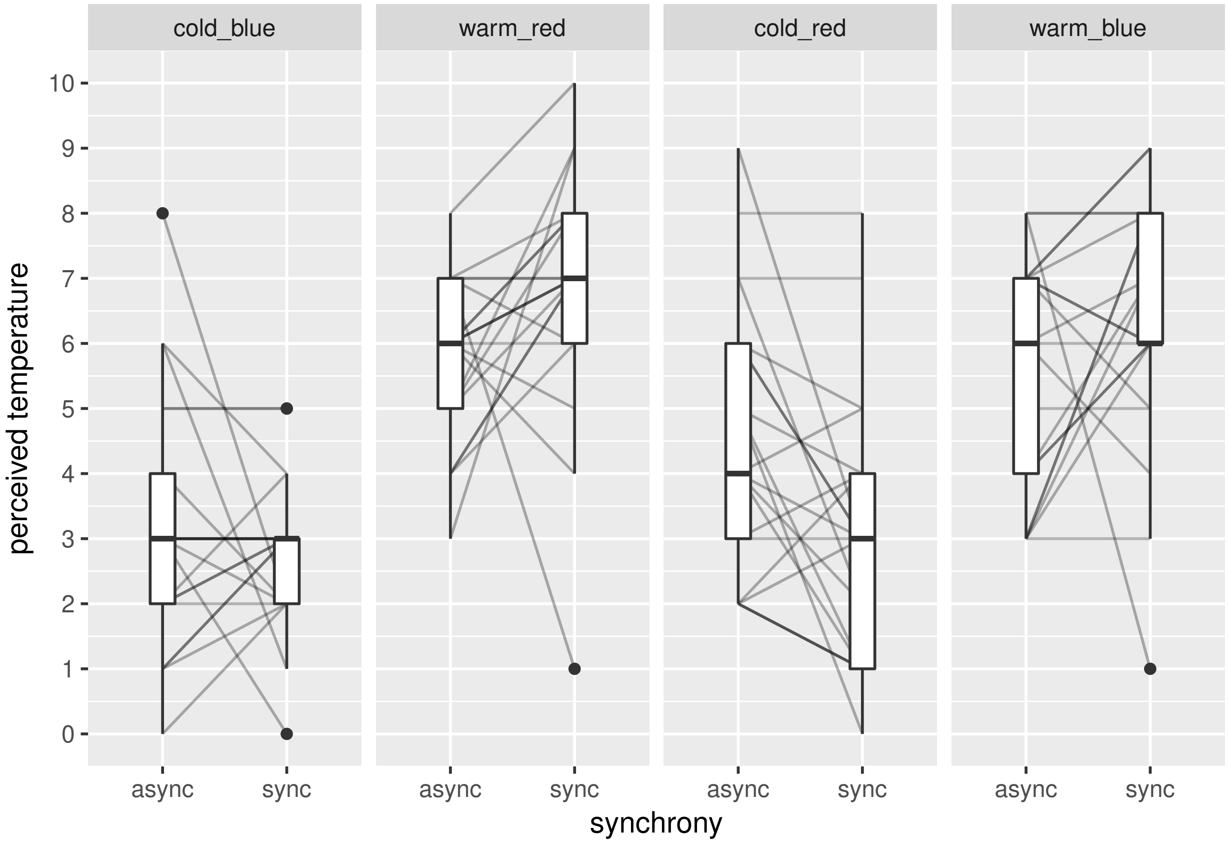

Barplot for publication

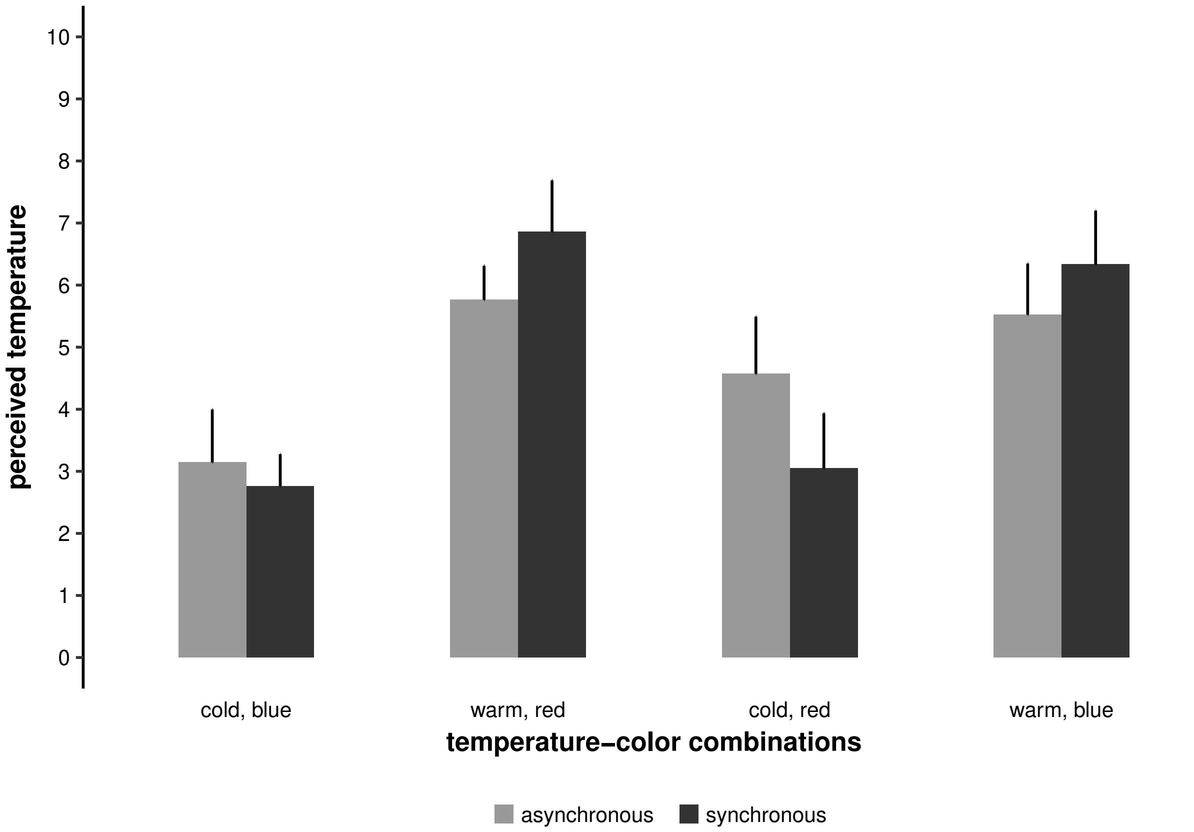

Linear Mixed Model

	Sum Sq	Mean Sq	NumDF	DenDF	F.value	Pr(>F)
synchrony	0.000000	0.000000	1	140	0.0000000	1.0000000
color	16.095238	16.095238	1	140	4.8666855	0.0290103
temperature	314.880952	314.880952	1	140	95.2099349	0.0000000
synchrony:color	1.928571	1.928571	1	140	0.5831384	0.4463698
synchrony:temperature	38.095238	38.095238	1	140	11.5187823	0.0008964
color:temperature	2.380952	2.380952	1	140	0.7199239	0.3976173
synchrony:color:temperature	5.357143	5.357143	1	140	1.6198288	0.2052252

Pairwise comparisons of the synchrony * temperature interaction using emmeans with FDR adjustment.

```
## Loading required namespace: pbkrtest
```

```
## NOTE: Results may be misleading due to involvement in interactions
## NOTE: Results may be misleading due to involvement in interactions
```

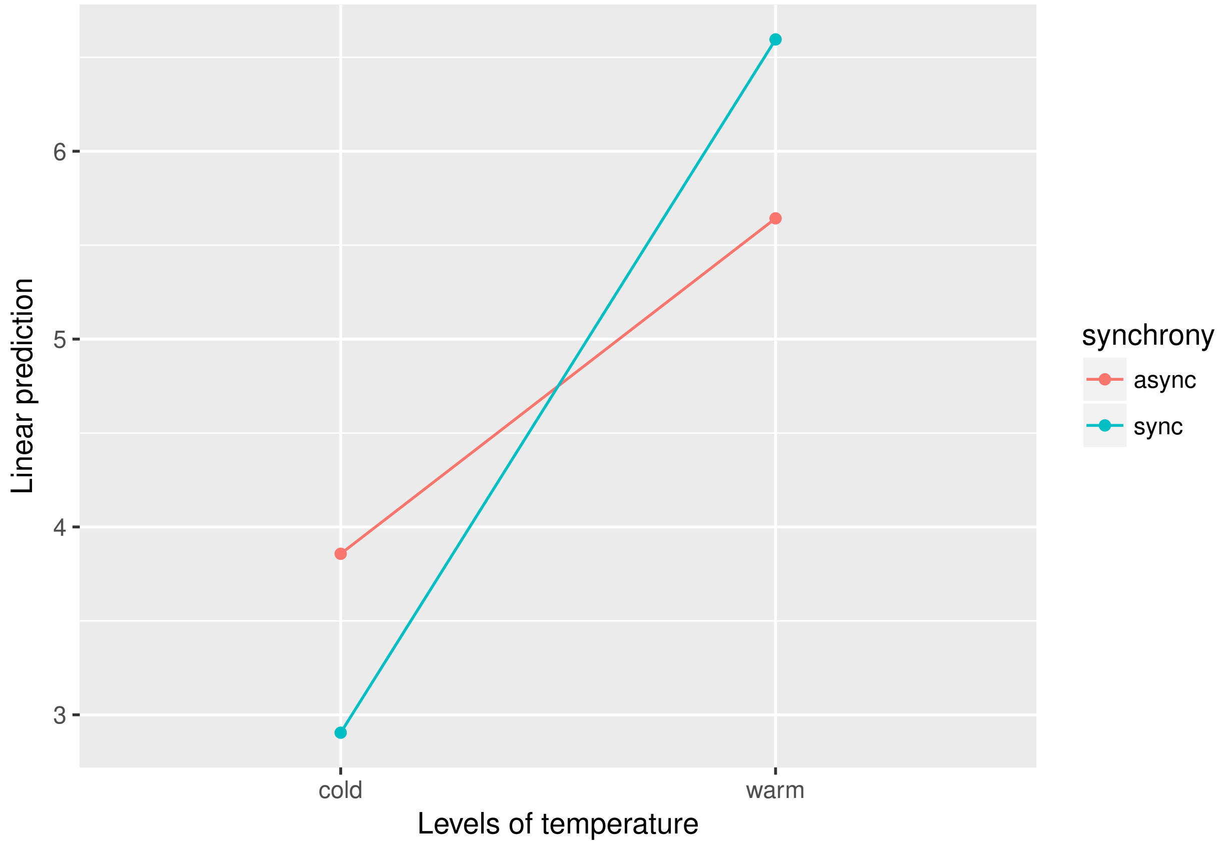

contrast	estimate	SE	df	t.ratio	p.value
async,cold - sync,cold	0.952381	0.3968464	140	2.399873	0.0177159
async,cold - async,warm	-1.785714	0.3968464	140	-4.499762	0.0000213
async,cold - sync,warm	-2.738095	0.3968464	140	-6.899635	0.0000000
sync,cold - async,warm	-2.738095	0.3968464	140	-6.899635	0.0000000
sync,cold - sync,warm	-3.690476	0.3968464	140	-9.299508	0.0000000
async,warm - sync,warm	-0.952381	0.3968464	140	-2.399873	0.0177159

Separate Linear Mixed Models for synchronous and asynchronous condition

	Sum Sq	Mean Sq	NumDF	DenDF	F.value	Pr(>F)
temperature	286.011905	286.011905	1	84	91.6984733	0.0000000
color	3.440476	3.440476	1	84	1.1030534	0.2966092
temperature:color	0.297619	0.297619	1	84	0.0954198	0.7581617

	Sum Sq	Mean Sq	NumDF	DenDF	F.value	Pr(>F)
temperature	66.964286	66.964286	1	63	23.666021	0.0000080
color	14.583333	14.583333	1	63	5.153933	0.0266237
temperature:color	7.440476	7.440476	1	63	2.629558	0.1098855

Vividness by synchrony and congruency

This is the analysis for our main hypothesis.

Boxplot

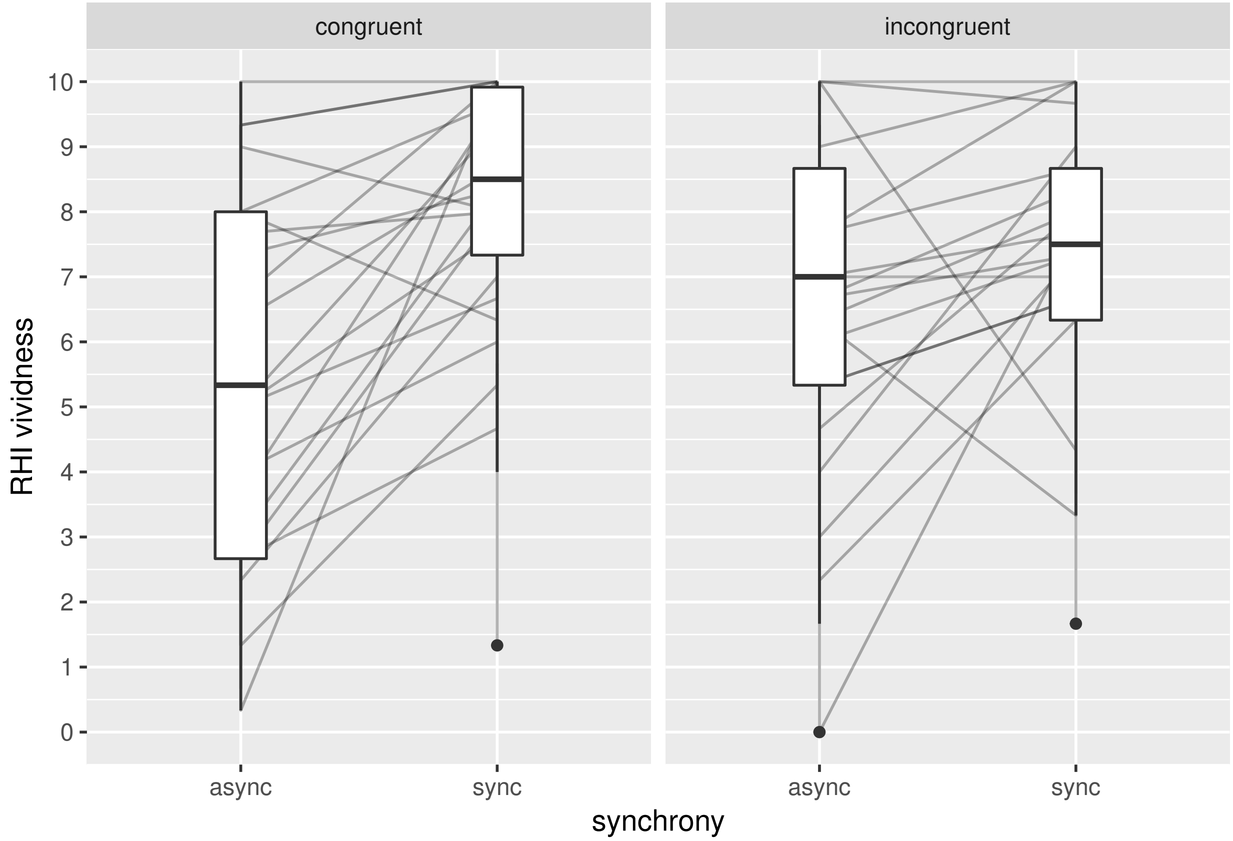

Barplot for publication

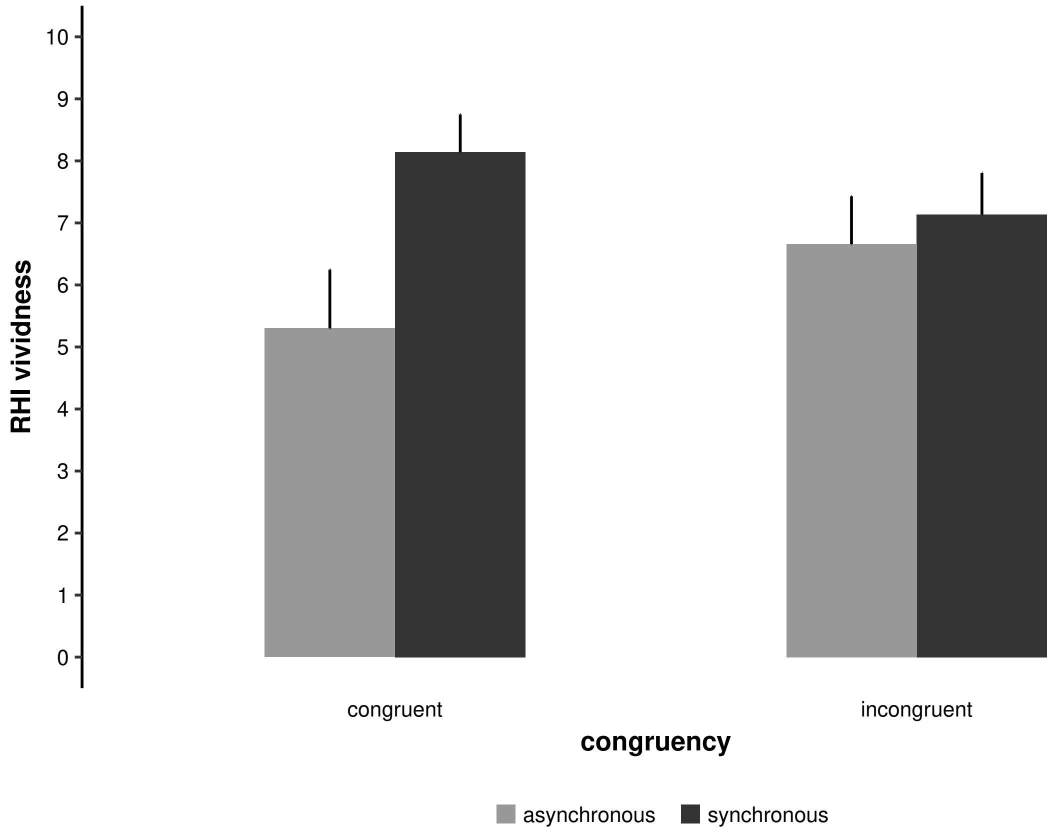

Linear Mixed Model

	Sum Sq	Mean Sq	NumDF	DenDF	F.value	Pr(>F)
synchrony	118.6817970	118.6817970	1	145.9676	33.397546	0.0000000
congruency	0.8969522	0.8969522	1	145.9676	0.252406	0.6161431
synchrony:congruency	55.6093667	55.6093667	1	145.9676	15.648705	0.0001186

Pairwise comparisons of the synchrony * congruency interaction using emmeans with FDR adjustment.

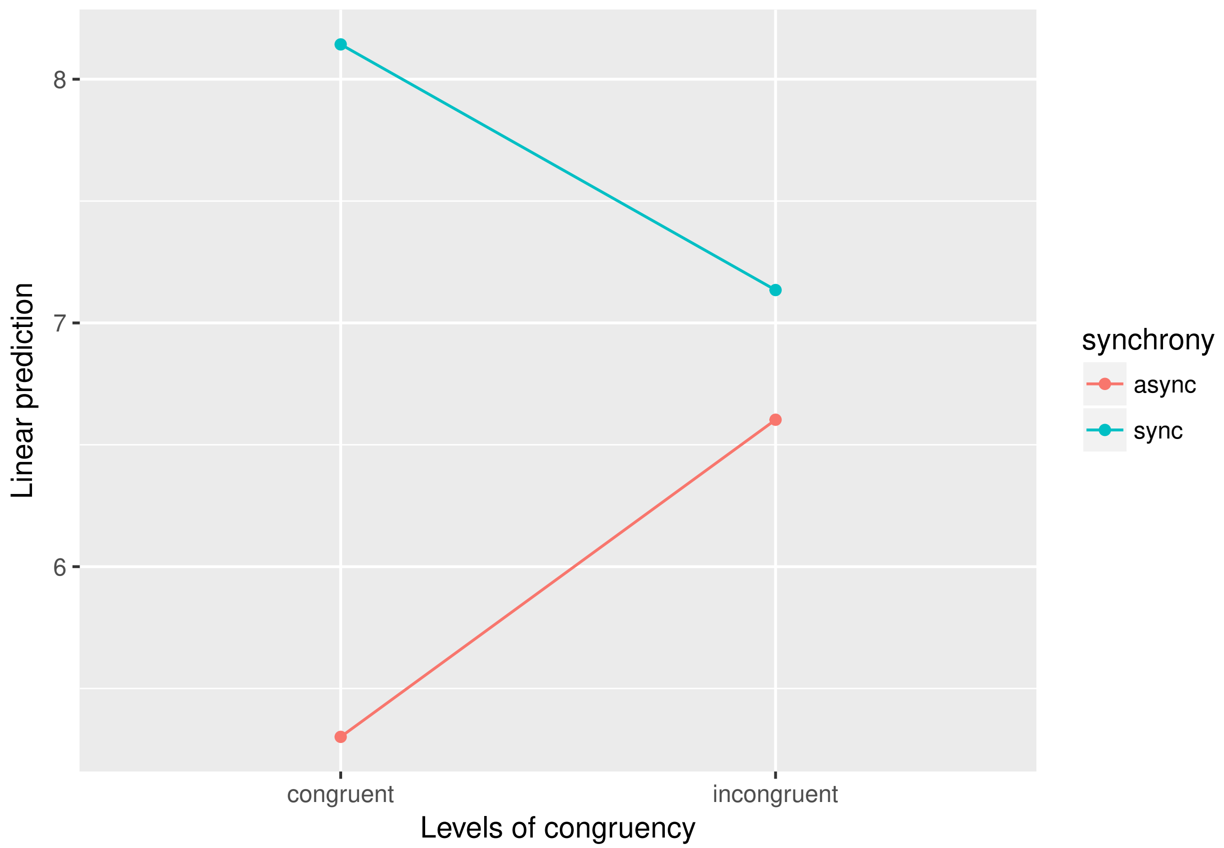

contrast	estimate	SE	df	t.ratio	p.value
async,congruent - sync,congruent	-2.8412698	0.4156520	149.0583	-6.835694	0.0000000
async,congruent - async,incongruent	-1.3012018	0.4184817	149.1442	-3.109339	0.0033698
async,congruent - sync,incongruent	-1.8333333	0.4156520	149.0583	-4.410741	0.0000589
sync,congruent - async,incongruent	1.5400681	0.4184817	149.1442	3.680132	0.0006501
sync,congruent - sync,incongruent	1.0079365	0.4156520	149.0583	2.424953	0.0198076
async,incongruent - sync,incongruent	-0.5321316	0.4184817	149.1442	-1.271577	0.2055030

Vividness by synchrony, color and temperature

Boxplot

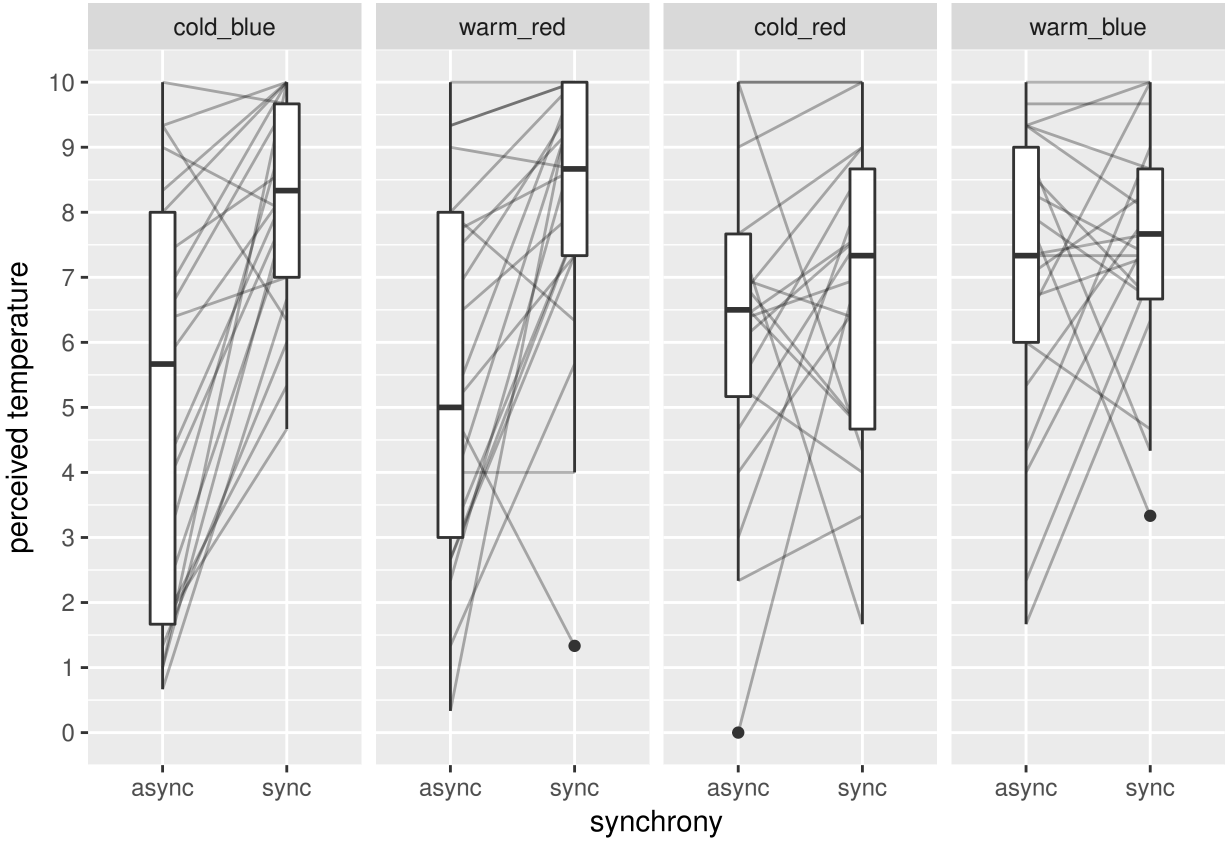

Barplot for publication

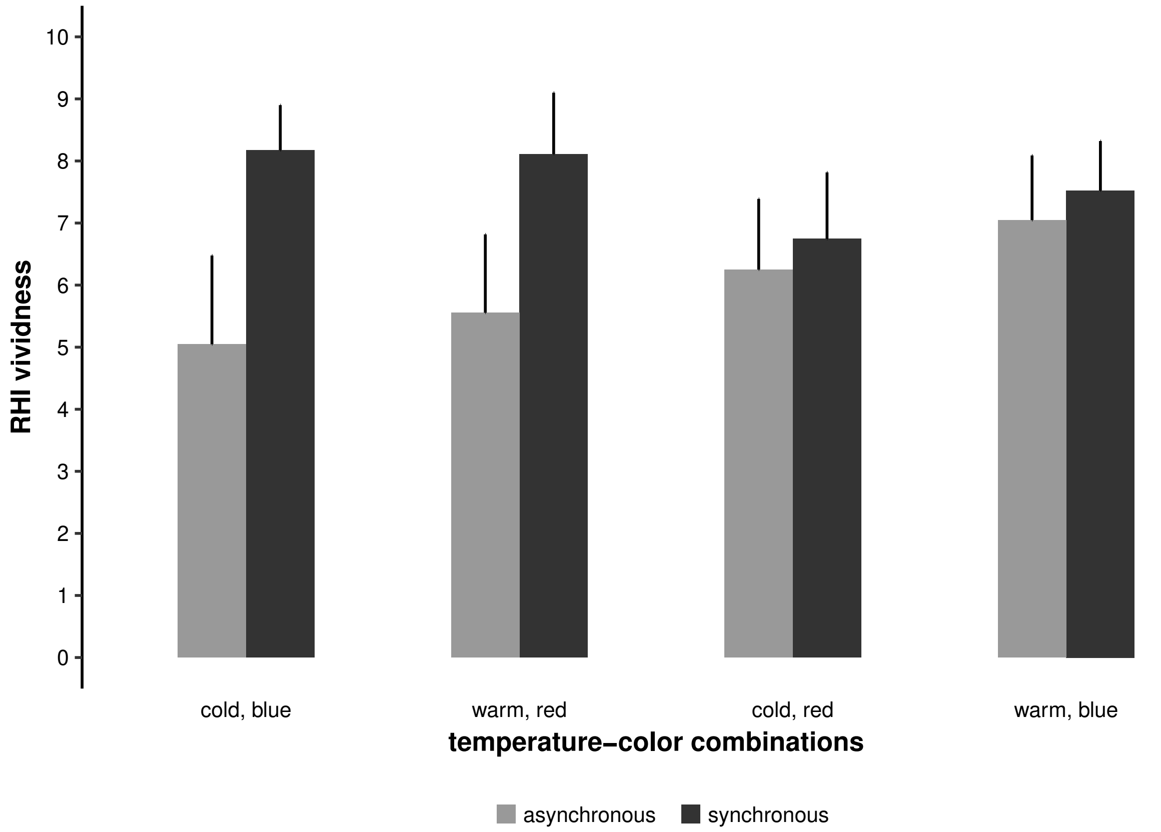

Linear Mixed Model

	Sum Sq	Mean Sq	NumDF	DenDF	F.value	Pr(>F)
synchrony	119.5700979	119.5700979	1	145.9632	34.8645553	0.0000000
color	4.0656265	4.0656265	1	145.9632	1.1854658	0.2780405
temperature	11.9128260	11.9128260	1	145.9632	3.4735723	0.0643654
synchrony:color	0.4902885	0.4902885	1	145.9632	0.1429596	0.7059057
synchrony:temperature	1.3110135	1.3110135	1	145.9632	0.3822687	0.5373552
color:temperature	0.8194384	0.8194384	1	145.9632	0.2389339	0.6257108
synchrony:color:temperature	54.9775887	54.9775887	1	145.9632	16.0305061	0.0000989

Separate Linear Mixed Models for synchronous and asynchronous condition

	Sum Sq	Mean Sq	NumDF	DenDF	F.value	Pr(>F)
temperature	2.678571	2.678571	1	62.99999	1.295099	0.2594199
color	3.715609	3.715609	1	62.99999	1.796510	0.1849488
temperature:color	21.334656	21.334656	1	62.99999	10.315382	0.0020789

	Sum Sq	Mean Sq	NumDF	DenDF	F.value	Pr(>F)
temperature	10.4085421	10.4085421	1	62.03995	3.0797289	0.0842108
color	0.8403796	0.8403796	1	62.03995	0.2486555	0.6197868
temperature:color	34.4269561	34.4269561	1	62.03995	10.1864114	0.0022214

Relationship between perceived temperature and RHI vividness

Scatter plots

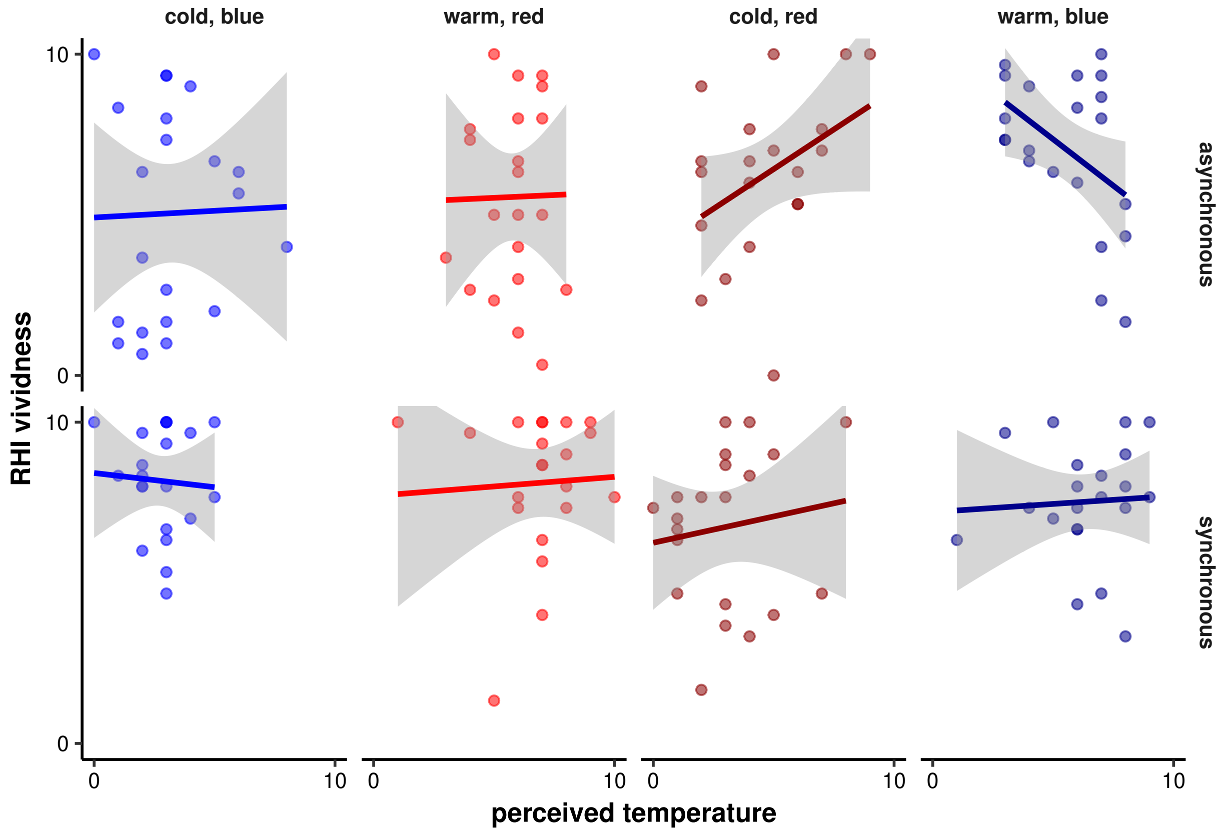

Regressions

```
## : async
## : cold
## : blue
##
## Call:
## lm(formula = rhi_ownership ~ perc_temp, data = x)
##
## Residuals:
##    Min     1Q Median     3Q    Max
## -4.333 -3.292  0.500  2.958  5.083
##
## Coefficients:
```

```
##             Estimate Std. Error t value Pr(>|t|)
## (Intercept)  4.91667    1.41165   3.483  0.00249 **
## perc_temp    0.04167    0.38386   0.109  0.91470
## ---
## Signif. codes:  0 '***' 0.001 '**' 0.01 '*' 0.05 '.' 0.1 ' ' 1
##
## Residual standard error: 3.359 on 19 degrees of freedom
## Multiple R-squared:  0.0006197,  Adjusted R-squared:  -0.05198
## F-statistic: 0.01178 on 1 and 19 DF,  p-value: 0.9147
##
## ------------------------------------------------------------
## : sync
## : cold
## : blue
##
## Call:
## lm(formula = rhi_ownership ~ perc_temp, data = x)
##
## Residuals:
##     Min      1Q  Median      3Q     Max
## -3.4869 -1.0651  0.0029  1.5811  2.0234
##
## Coefficients:
##             Estimate Std. Error t value     Pr(>|t|)
## (Intercept)  8.41895    0.96560   8.719 0.0000000456 ***
## perc_temp   -0.08847    0.32272  -0.274        0.787
## ---
## Signif. codes:  0 '***' 0.001 '**' 0.01 '*' 0.05 '.' 0.1 ' ' 1
##
## Residual standard error: 1.702 on 19 degrees of freedom
## Multiple R-squared:  0.00394,    Adjusted R-squared:  -0.04848
## F-statistic: 0.07515 on 1 and 19 DF,  p-value: 0.7869
##
## ------------------------------------------------------------
## : async
## : warm
## : blue
##
## Call:
## lm(formula = rhi_ownership ~ perc_temp, data = x)
##
## Residuals:
##     Min      1Q  Median      3Q     Max
## -3.9492 -1.1736 -0.5069  1.5611  3.8059
##
## Coefficients:
##             Estimate Std. Error t value   Pr(>|t|)
## (Intercept)  10.2415     1.5017   6.820 0.00000165 ***
## perc_temp    -0.5782     0.2579  -2.242     0.0371 *
## ---
## Signif. codes:  0 '***' 0.001 '**' 0.01 '*' 0.05 '.' 0.1 ' ' 1
##
## Residual standard error: 2.177 on 19 degrees of freedom
## Multiple R-squared:  0.2092, Adjusted R-squared:  0.1676
```

```
## F-statistic: 5.027 on 1 and 19 DF,  p-value: 0.03709
##
## ------------------------------------------------------------
## : sync
## : warm
## : blue
##
## Call:
## lm(formula = rhi_ownership ~ perc_temp, data = x)
##
## Residuals:
##     Min      1Q  Median      3Q     Max
## -4.2752 -0.8402  0.0073  1.1598  2.5440
##
## Coefficients:
##             Estimate Std. Error t value  Pr(>|t|)
## (Intercept)  7.20178    1.39783   5.152 0.0000567 ***
## perc_temp    0.05085    0.21107   0.241     0.812
## ---
## Signif. codes:  0 '***' 0.001 '**' 0.01 '*' 0.05 '.' 0.1 ' ' 1
##
## Residual standard error: 1.872 on 19 degrees of freedom
## Multiple R-squared:  0.003045,   Adjusted R-squared:  -0.04943
## F-statistic: 0.05803 on 1 and 19 DF,  p-value: 0.8122
##
## ------------------------------------------------------------
## : async
## : cold
## : red
##
## Call:
## lm(formula = rhi_ownership ~ perc_temp, data = x)
##
## Residuals:
##     Min      1Q  Median      3Q     Max
## -6.4229 -1.5835  0.1635  1.6324  4.0589
##
## Coefficients:
##             Estimate Std. Error t value Pr(>|t|)
## (Intercept)   3.9532     1.3438   2.942  0.00872 **
## perc_temp     0.4939     0.2638   1.872  0.07749 .
## ---
## Signif. codes:  0 '***' 0.001 '**' 0.01 '*' 0.05 '.' 0.1 ' ' 1
##
## Residual standard error: 2.454 on 18 degrees of freedom
##   (1 observation deleted due to missingness)
## Multiple R-squared:  0.163,  Adjusted R-squared:  0.1165
## F-statistic: 3.506 on 1 and 18 DF,  p-value: 0.07749
##
## ------------------------------------------------------------
## : sync
## : cold
## : red
##
```

```
## Call:
## lm(formula = rhi_ownership ~ perc_temp, data = x)
##
## Residuals:
##     Min      1Q  Median      3Q     Max
## -4.9078 -2.4049  0.9284  1.9284  3.2618
##
## Coefficients:
##             Estimate Std. Error t value   Pr(>|t|)
## (Intercept)   6.2468     0.9954   6.275 0.00000503 ***
## perc_temp     0.1638     0.2736   0.599      0.556
## ---
## Signif. codes:  0 '***' 0.001 '**' 0.01 '*' 0.05 '.' 0.1 ' ' 1
##
## Residual standard error: 2.492 on 19 degrees of freedom
## Multiple R-squared:  0.01852,    Adjusted R-squared:  -0.03314
## F-statistic: 0.3584 on 1 and 19 DF,  p-value: 0.5565
##
## --------------------------------------------------------
## : async
## : warm
## : red
##
## Call:
## lm(formula = rhi_ownership ~ perc_temp, data = x)
##
## Residuals:
##     Min      1Q  Median      3Q     Max
## -5.2655 -2.5639 -0.5289  2.4012  4.4711
##
## Coefficients:
##             Estimate Std. Error t value Pr(>|t|)
## (Intercept)  5.35429    3.09847   1.728    0.100
## perc_temp    0.03493    0.52589   0.066    0.948
##
## Residual standard error: 2.966 on 19 degrees of freedom
## Multiple R-squared:  0.0002321,  Adjusted R-squared:  -0.05239
## F-statistic: 0.004412 on 1 and 19 DF,  p-value: 0.9477
##
## --------------------------------------------------------
## : sync
## : warm
## : red
##
## Call:
## lm(formula = rhi_ownership ~ perc_temp, data = x)
##
## Residuals:
##     Min      1Q  Median      3Q     Max
## -6.6669 -0.7266  0.5470  1.7609  2.2386
##
## Coefficients:
##             Estimate Std. Error t value Pr(>|t|)
## (Intercept)  7.70166    1.93690   3.976 0.000809 ***
```

```
## perc_temp    0.05971    0.27262   0.219 0.828967
## ---
## Signif. codes:  0 '***' 0.001 '**' 0.01 '*' 0.05 '.' 0.1 ' ' 1
##
## Residual standard error: 2.322 on 19 degrees of freedom
## Multiple R-squared:  0.002518,   Adjusted R-squared:  -0.04998
## F-statistic: 0.04797 on 1 and 19 DF,  p-value: 0.829
```
